# Increase in electron scattering length in PEDOT:PSS by a triflic acid post-processing

**DOI:** 10.1007/s00706-017-1973-1

**Published:** 2017-03-31

**Authors:** Dominik Farka, H. Coskun, P. Bauer, D. Roth, B. Bruckner, Petr Klapetek, N. Serdar Sariciftci, P. Stadler

**Affiliations:** 1 Linz Institute for Organic Solarcells (LIOS), Institute of Physical Chemistry, Johannes Kepler University Linz, Altenberger Strasse 69, 4040 Linz, Austria; 2 Department of Physics, Atom and Surface Physics, Johannes Kepler University Linz, Altenbergerstr. 69, 4040 Linz, Austria; 3 Department of Nanometrology, Czech Metrology Institute, Okružní 31, 63800 Brno, Czech Republic

**Keywords:** Transparent conductive electrodes, Conductive metallic polymers, Anderson localization, Mott–Ioffe–Regel limit, Infrared transparency

## Abstract

**Abstract:**

A stringent limitation in many optoelectronic devices, such as solar cells and light emitting diodes, is the intrinsic need for a transparent electrode. Uniting relevant aspects, indium tin oxide (ITO) is often the material of choice, however, alternatives are sought and being in particular found in conductive polymers. In this work, we present a novel doping strategy to arrive at highly conducting polymeric material based on poly-3,4-ethylenedioxythiophene (PEDOT). Based on commercial high conductivity PEDOT:PSS (Clevios PH 1000), and a post processing with aqueous triflic acid delivers a material that is both transparent and of low resistivity (5.23 × 10^−4^ Ω cm). Furthermore, this material retains its conductive character over a large temperature range, indicating metallic behaviour. This is further supported by positive magnetoconductance effects at low temperatures (1.8–10 K) and extended mean free paths of the conduction electrons are observed—evidencing for a metallic state in this polymer.

**Graphical abstract:**

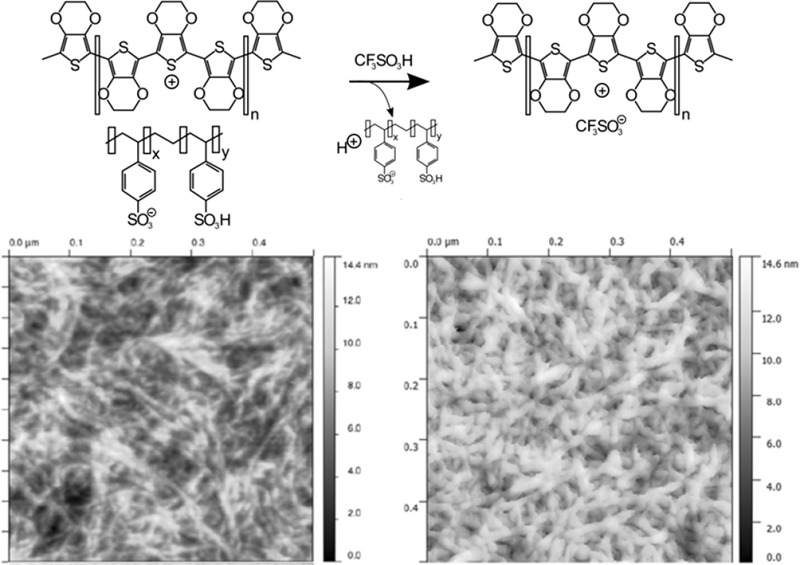

**Electronic supplementary material:**

The online version of this article (doi:10.1007/s00706-017-1973-1) contains supplementary material, which is available to authorized users.

## Introduction

In recent years, the rise of consumer electronics with liquid crystal displays, organic light emitting diode (OLED)-based displays [1], OLED-based home lighting [2], and the introduction of concepts such as photovoltaic windows [3] led to a dramatic increase in demand for transparent electrode materials. This demand led to a substantial price-increase of the current state-of-the-art material, indium-doped tinoxide (ITO) [4].

An alternative to inorganic oxides for transparent electrodes can be found in organic, conductive polymers. The primary advantage of organic polymers lies in their reliance on organic synthesis, opening a limitless multitude of possible structures, virtually only limited by the researchers’ fantasy. Chemically or electrochemically doping of an organic, conjugated polymer then delivers a conducting material from a semiconducting (or even insulating) starting system. This doping process, however, introduces disorder into the system—as opposed to inorganic semiconductors, where doping process replaces atoms. Organic molecules and counterions need to move inside the film, introducing disorder and thereby quenching the materials metallic properties [5–7].

In recent years, doped poly-3,4-ethylenedioxythiophene (PEDOT) has become the material of choice in the industry among the transparent, organic conductors, and many researchers have combined their efforts in its development [8–13]. To date, the commercially available doped PEDOT polymer combines both, good conductivity and transparency, it has found use in various types of devices and applications [14–19].

In this publication, we present a method to increase the conductivity of commercially available PEDOT:PSS by counter-ion exchange. Based on commercial Clevios PH1000 (high conductivity PEDOT:polystyrene sulfonate), a comparison between the conductivity of the material arrived at by spin-coating the dispersion as is (referred to as PEDOT:PSS), with addition of DMSO (5% by volume; PEDOT:PSS*), and with additional processing by triflic acid (PEDOT:TA) exposure will be comparatively presented. Furthermore, we investigated the correlation between PEDOT-content and resistivity in aforementioned systems. The enhanced metallic character of PEDOT:TA made magnetotransport measurements possible at low temperatures.

## Results and discussion

**Fig. 1:**
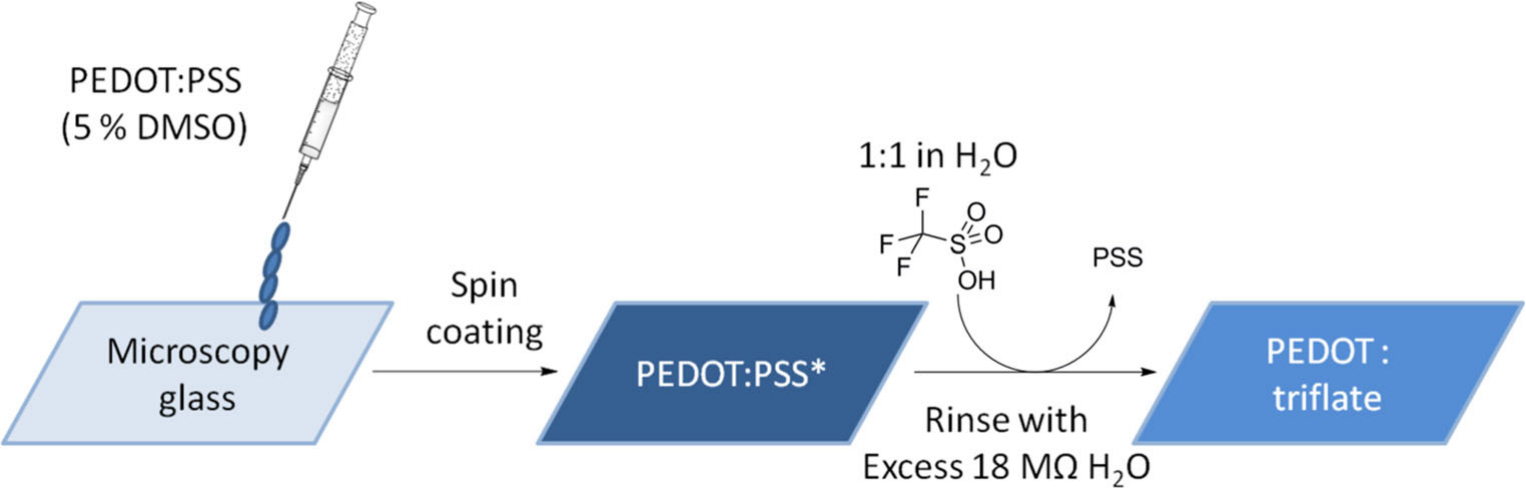
Preparation of PEDOT: triflate films via solution processing

Relying solely on solution processing, the herein presented films were spin-coated on top of glass or sapphire (1110) substrates. The schematics of film preparation can be found in Fig. 1. In this study, we compared three materials based on commercial PEDOT:PSS (Clevios PH1000): plain PEDOT:PSS (obtained from untreated dispersion), PEDOT:PSS* (obtained from a dispersion with 5% DMSO content), and PEDOT:TA (the latter substance treated with excess of equimolar, aqueous triflic acid, neutralized by rinsing with excess 18 MΩ cm water). As the exposure to triflic acid leads to dramatic decrease in film thickness, multiple-layers of PEDOT were spin-coated to achieve films of thicknesses similar to other materials.

**Fig. 2:**
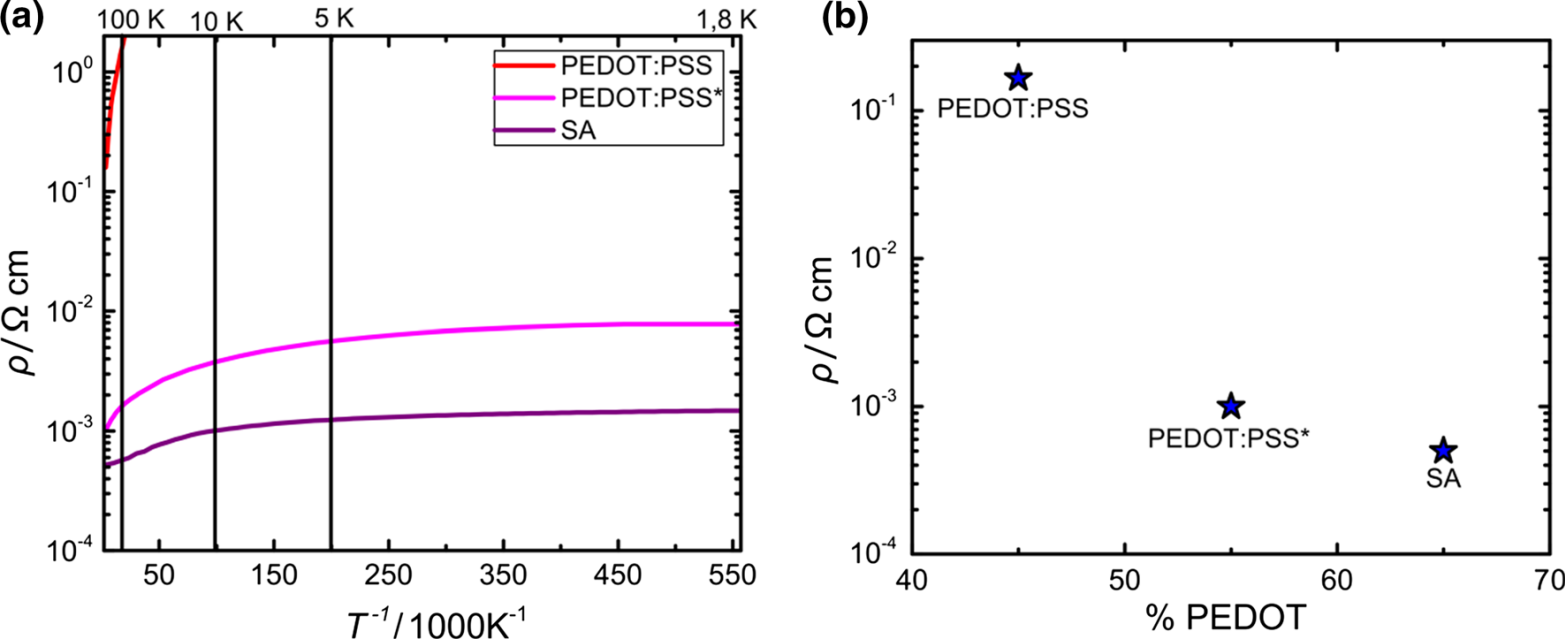
**a** Comparison of resistivity profiles of PEDOT derivatives over a temperature range between 300 and 1.8 K. Note the dramatic decrease in PEDOT:PSS. A heavy reliance on thermally activated transport is required. **b** Correlation of PEDOT-content and conductivity aforementioned PEDOT derivatives. An exponential drop in resistivity (on a log-scale) with the content of conducting material within the film can be observed

To compare resistivity, a number of versions of PEDOT were processed on top of sapphire samples containing previously deposited electrodes (Cr/Au, 8 nm/80 nm, respectively) in van-der-Pauw and four-in-line geometries. In this way, artefacts originating from geometric effects were minimized. A thorough resistivity-scan over a wide range of temperatures was performed. The results can be found in Fig. 2a.

As expected, samples obtained from spin-coating the as-is dispersion showed a strongly expressed dependence on temperature (PEDOT:PSS), the films becoming virtually insulating upon cooling. This points towards a purely, temperature-activated process requiring a high activation energy of 8.6 meV. The DMSO-treated samples (PEDOT:PSS*) showed substantially better resistivity dependence on temperature, albeit overshadowed by the performance of PEDOT:TA.

The dynamics during spin-coating deposition are quite complex, since varying turning speeds and tuning the DMSO concentration effect substantially different film properties. One role we attribute to DMSO is the improvement of the morphology of the resulting PEDOT:PSS films by acting as a co-solvent. The second contribution is the improvement of film purity as shown by RBS results. This is true regarding the stoichiometry between conducting polymer and doping agent as well as for the removal of other impurities (see Supplementary Material Figs. 1 and 2, respectively).

Since DMSO is a good solvent (polar and aprotic) it dissolved impurities and excess PSS, leaving behind a 1:1 ratio of PEDOT:PSS with diminished impurities (see Supplementary Material Figs. 1 and 2, respectively). With decreasing temperature, PEDOT:PSS* electron-transport properties worsened by a full order of magnitude. This hints to a metallic behaviour within this material, and can be seen as low resistivity at low temperatures rather unusual for a conductive polymer—probably due to the advantageous effects of DMSO mentioned above [20, 21].

Strikingly, the material receiving triflic acid-treatment still showed even lower resistivities at temperatures below 2 K, namely 1.4 × 10^−3^ Ω cm—a more than fivefold improvement over PEDOT:PSS*. This overall low resistivity of PEDOT:TA and good retention of performance over a large temperature range indicate enhanced order within the film meaning that the disorder introduced during the anion-exchange is comparably small, its adverse effects by far outweighed by the positive ones. This picture, however, does not include the possibility of a correlation between enhanced electro-transport and the amount of conductive material within the film.

To be able to exclude the possibility of a sole “concentration effect” of conductor versus counterion [21], the content of PEDOT (by molecular %) within the sample was investigated using Rutherford Backscattering (RBS, see SI for original data). From that, the PEDOT-content within the film was derived. The results of the comparison are shown in Fig. 2b.

The results clearly indicate a partial dependence of PEDOT content. As expected, a large excess of PSS in PEDOT:PSS correlated well with its electrical performance at room temperature. Truly, an increased amount of PEDOT within the triflate-treated films was found, over PEDOT:PSS*. A 20% increase in content of conducting material over PEDOT:PSS* within the thin-films resulted in a de facto halving of the resistivity at room temperature.

**Fig. 3:**
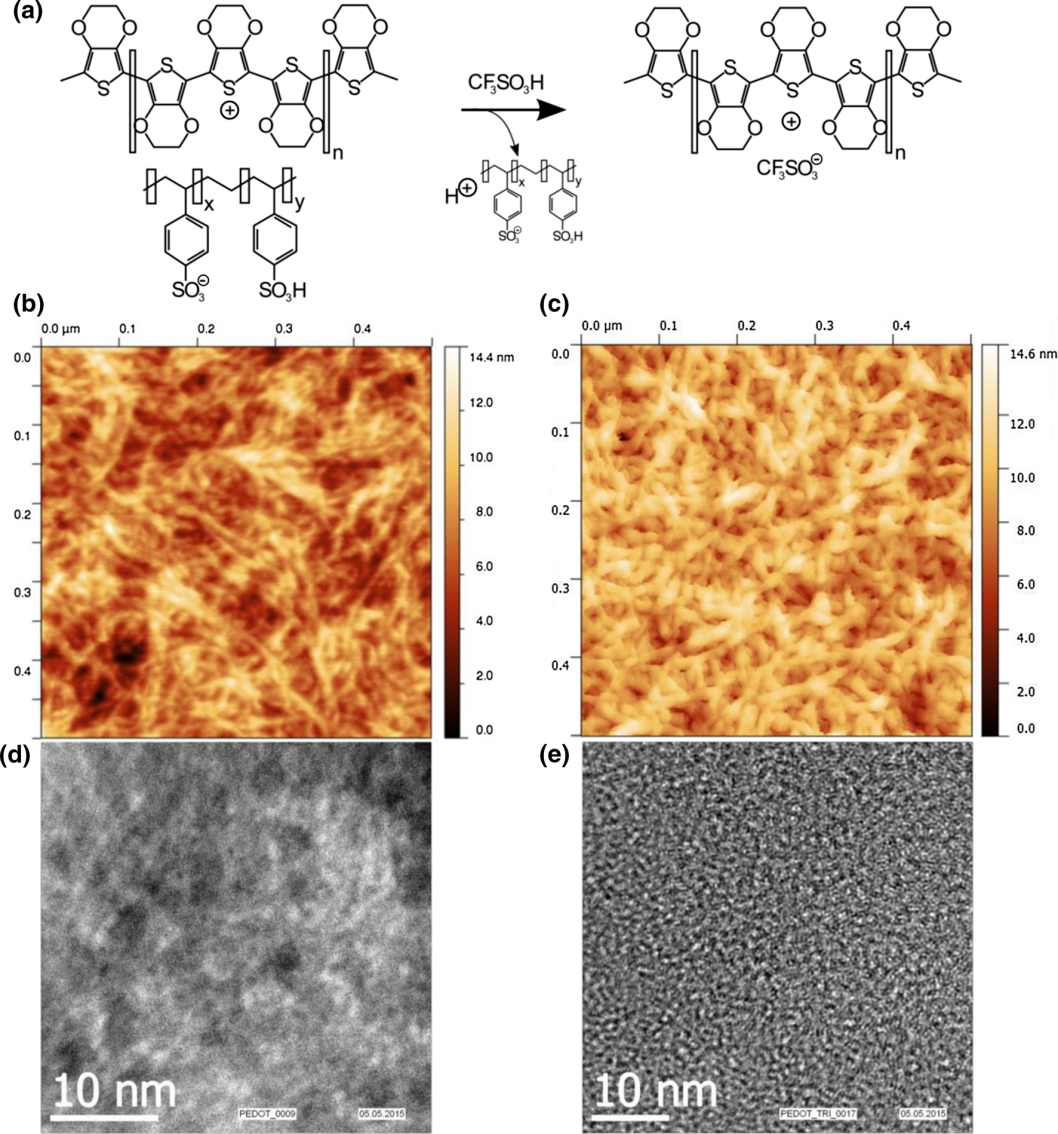
**a** Counterion exchange mechanism. **b** AFM image of PEDOT:PSS* on a 500 × 500 nm area compared to PEDOT:TA on the same scale. **c** Note the decrease in roughness as fewer voids are left within the film, corresponding to enhanced electro-transport properties. **d**, **e** Comparison of TEM images of these films. A more uniform distribution of moieties within the film can be observed, correlating well with the AFM results

To further strengthen the argument of order over “concentration”, Atomic Force Microscopy (AFM) was performed on PEDOT:PSS* (Fig. 3b) and PEDOT:TA (Fig. 3c). It appeared that the global order within the film remained similar, yet smoother films with fewer depressions were obtained. In Fig. 3d and e, we compare the same films via Transmission Electron Microscopy (TEM). Also there, an increase of order in the form of a more uniform distribution of moieties within the film can be observed after acid-treatment.

However, no global connectivity was observed in either case—even worse, a picture of many separate grains was observed. Hence, it seemed feasible, that the increased conductivity is indeed mainly caused by the elevated PEDOT-content.

**Fig. 4:**
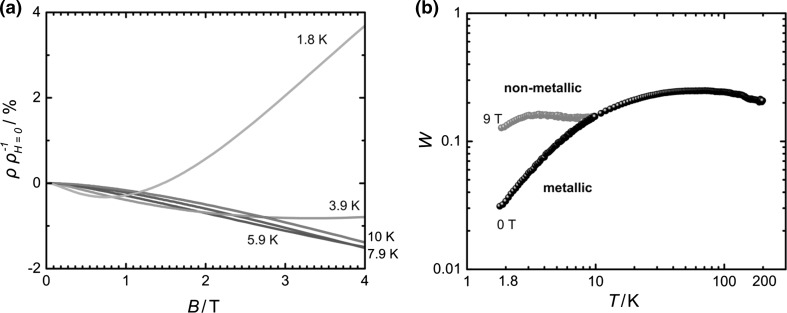
**a** Magnetoconductivity of PEDOT:TA. The minima 0.74 and 3.4 T correspond to mean free electron paths of 58.4 and 27.2 nm. **b** Zabrodskii diagrams of PEDOT:TA. The so-called *W*-plot describes a change from an Anderson insulator to a glassy metal [22]. The application of a magnetic field of 9 T at 1.8 K effects a disturbance within the materials way of conduction, thus leading destroying the metallic properties within the material

As PEDOT:TA already showed metallic fingerprints in the form of a rather low and flat resistivity-temperature profile we looked for magnetoresistance behaviour at low temperatures, typical for metals. As such effects are expected to be most pronounced at low temperatures, scans at 1.8, 3.8, 5.9, 7.9, and 10 K were performed at fields ranging between 0 and 9 T (see Fig. 4a). At the lowest two temperatures, 1.8 and 3.8 K, the described effect is best visible. At first, a negative magnetoresistance can be observed at lower fields (up to 0.74 and 3.4 T, respectively).

On the contrary, upon trespassing this threshold, positive magnetoresistance was observed as the metallic behaviour was disordered by the field. Having both contributions is a hallmark of metallic polymers. From that, it is possible to calculate the Landau orbit size, *L*_D_, which corresponds to the magnetic penetration depth. In that way, the electron scattering length, *λ*_ε_, can be derived (Eq. 1).

1 $$L_{\text{D}} = \lambda_{\varepsilon } \quad L_{\text{D}} = \sqrt {\frac{\hbar }{e \cdot B}}$$

For the obtained fields, the corresponding electron scattering length at 1.8 K corresponds to 58.4 nm and to 27.2 nm for 3.9 K. These are truly astonishing findings for a conducting polymer, especially in comparison with values reported earlier for PEDOT:PSS* [7]. As high PEDOT-content alone does not explain this effect local order of the polymer appears to be improved to explain such behaviour.

As the measurement of magnetoconductivity was possible, we were further interested, to see the difference between the resistivity profile when exposed to a strong magnetic field (9 T) and none (0 T). The results of such a measurement can be plotted in the so-called *W*-plot, reported by Zabdrodskii and Zinojeva [22]. Here, log *T* is plotted versus the negative value of the first derivative of resistivity after temperature, helping to visualize the type of transport of the material of question.

2 $$W = - \left( {\frac{{\delta { \ln }\rho }}{{\delta { \ln }T}}} \right)$$

*W* is used to expand the low-*T* transition regime between critically metallic behaviour $\left( {\frac{\Delta W}{\Delta T}\sim {\text{const}}.} \right)$ and actually metallic (*W* < 0). The log–log plot in Fig. 4b exactly shows such a trend in PEDOT:TA, i.e. *W* exponentially approaches 0 with decreasing temperature characteristic for Anderson transitions in (metallic) conductive polymers.

As one can clearly see, at the absence of a magnetic field, the material clearly behaves as a glassy metal, getting closer to a metallic behaviour with decreasing temperature. This would indicate that order is induced with cooling the sample, supporting metallic transport modes. Repeating the same experiment at a high field (9 T), a different behaviour is observed. When cooling the sample, a flat profile for *W* versus *T* is observed, this means that the metallic features are disrupted by the strong external magnetic field. All those effects are strong indications for a metallic state in this polymer, which was achieved by anion exchange and solution processing.

## Conclusion

Three different solution-processed, PEDOT-based conducting materials were compared for their resistivity. In the case of PEDOT:TA (triflate), a flat temperature profile of resistivity down to 1.8 K is observed, indicating metallic behaviour. A correlation between the materials content of conducting polymer and the resistivity was observed. Structural film-analysis via AFM and SEM indicated no global order within the film. Rough and globally disordered films were obtained upon treatment with triflic acid. Those films, however, showed a positive magnetoresistance, typical for metals. Also, at low temperatures (1.8 and 3.9 K), tremendous values for mean free path of electrons conducted by PEDOT:TA were found, ranging in the hundreds of Angstroms implying local order within the material. This was further supported by the results of the *W*-plots, where the metallic properties were quenched by applying a strong magnetic field, implying a metallic state.

## Experimental

### Substrate preparation

Sapphire (polished, 1110) and glass (for AFM measurements) substrates were used as substrates for all experiments. For cleaning, a four-step washing procedure (15 min steps) by consecutive sonication in different solvents was conducted: acetone (technical grade, room temperature), 2-propanol (50 °C), Hellmanex detergent (70 °C), and deionized water (room temperature). The electrical contacts were deposited using PVD through the van-der-Pauw and four-in-line mask (8 nm Cr/80 nm Au), respectively.

### Conductive polymer deposition

All samples were prepared using spin-coating. PEDOT:PSS thin-films were achieved by spin-coating of commercial Clevios PH1000 (a dispersion) as obtained from the manufacturer (Heraeus) on top of the respective substrate. PEDOT:PSS* and PEDOT:TA thin-films were both prepared in the following way: 5% DMSO (v/v) was added to dispersions of Clevios PH1000 (by the Heraeus company), freshly before spin-coating. For all spin-coating steps the same recipe was used (recipe: 10 rps, 2 s ramp, 30 s spinning; 100 rps, 2 s ramp, 30 s spinning).

To achieve PEDOT:TA, the prepared sample was exposed to an excess of an equimolar solution of triflic acid in water for 1 min followed by three consecutive steps of exposure to an excess 18 MΩ cm water. After exposure, the samples were dried by spin-coating using the same recipe as mentioned above. For conductivity measurements, the active area of the samples measured was protected by drop-casting PMMA (in anhydrous toluene) on top of the measured area. Coating was performed on top of sapphire substrates and microscopy glass slides. For TEM measurements, the same procedure was applied on top of a copper-grid.

### Film characterization

AFM data were obtained using Dimension Icon SPM from Bruker in ScanAsyst regime which is an intermittent contact measurement procedure suitable for soft samples. Scanasyst-Air probes were used, with nominal probe radius in range of 2–12 nm. Scanning speed was approximately 0.2 Hz (for individual fast scan axis profiles). All TEM measurements were done using a JEOL JEM-2011. The samples (PEDOT:PSS* and PEDOT:TA) were deposited on top of a copper grid as described above. All pictures shown in this publication were obtained using the same focus and a voltage of 100 kV.

Rutherford backscattering spectrometry (RBS) measurements were performed at the Department of Atomic Physics and Surface Science (JKU) using the AN-700 van de Graaff accelerator in a HV chamber (base pressure in the 10^−7^ mbar range), which is equipped with two semiconductor surface barrier (SSB) detectors: a LN_2_-cooled high resolution detector [23] situated at 150.1° in Cornell geometry (FWHM ~3 keV), and a standard SSB detector of larger solid angle in 154.6° in IBM geometry. Energy spectra of the PEDOT:PSS samples on Si were recorded using 200 keV D^+^ ions and normal incidence of the ion beam, for the PEDOT:PSS samples on sapphire 220 keV D^+^ ions and an angle of incidence of 60° were used to optimize depth resolution. To avoid charging effects due to sapphire substrate, prior to the RBS measurements the samples were coated with a thin gold layer. The experimental spectra were evaluated employing the SIMNRA simulation software [24]; the uncertainty of the final compositions is ~10–20% with higher values for the samples on sapphire substrates.

The PMMA-covered PEDOTs were contacted using indium solder and loaded to the magnetotransport system (DynaCool PPMS, QuantumDesign and Lakeshore 8040 series, respectively). The electrical resistivity *ρ*_*xx*_ was characterized as function of temperature between 300 and 1.8 K. A reproduction in the Lakeshore system between 300 and 10 K was done using the van-der-Pauw electrode-geometry. In the Dynacool system, magnetoresistance measurements were conducted at fields between 0 and 9 T for following temperatures: 1.8, 3.8, 5.9, 7.9, and 10 K.

## Electronic supplementary material

Below is the link to the electronic supplementary material.

Supplementary material 1 (DOCX 796 kb)

## References

[1] Tsujimura T (2012). OLED displays: fundamentals and applications.

[2] Eritt M, May C, Leo K, Toerker M, Radehaus C (2010). Thin Solid Films.

[3] Zhang W, Lu L, Peng J, Song A (2016). Energy Build.

[4] Meiss J, Uhrich CL, Fehse K, Pfuetzner S, Riede MK, Leo K (2008). Transparent electrode materials for solar cells. SPIE Proc.

[5] Coclite AM, Howden RM, Borrelli DC, Petruczok CD, Yang R, Yagüe JL, Ugur A, Chen N, Lee S, Jo WJ, Liu A, Wang X, Gleason KK (2013). Adv Mater.

[6] Kang K, Watanabe S, Broch K, Sepe A, Brown A, Nasrallah I, Nikolka M, Fei Z, Heeney M, Matsumoto D, Marumoto K, Tanaka H, Kuroda S, Sirringhaus H (2016). Nat Mater.

[7] Stadler P, Farka D, Coskun H, Głowacki ED, Yumusak C, Uiberlacker LM, Hild S, Leonat LN, Scharber MC, Klapetek P, Menon R, Sariciftci NS (2016). J Mater Chem C.

[8] Du X, Wang Z (2003). Electrochim Acta.

[9] Perepichka IF, Levillain E, Roncali J (2004). J Mater Chem.

[10] Greczynski G, Kugler T, Keil M, Osikowicz W, Fahlman M, Salaneck WR (2001). J Electron Spectros Relat Phenom.

[11] Farah A, Rutledge S, Schaarschmidt A, Lai R, Freedman JP, Helmy AS (2012). J Appl Phys.

[12] Casado N, Hernández G, Veloso A, Devaraj S, Mecerreyes D, Armand M (2016). ACS Macro Lett.

[13] Jönsson SKM, Birgerson J, Crispin X, Greczynski G, Osikowicz W, Denier van der Gon AW, Salaneck WR, Fahlman M (2003). Synth Met.

[14] Bandodkar AJ, Nuñez-Flores R, Jia W, Wang J (2015). Adv Mater.

[15] Okuzaki H, Suzuki H, Ito T (2009). Synth Met.

[16] Lang U, Rust P, Dual J (2008). Microelectron Eng.

[17] Kaltenbrunner M, Adam G, Głowacki ED, Drack M, Schwödiauer R, Leonat L, Apaydin DH, Groiss H, Scharber MC, White MS, Sariciftci NS, Bauer S (2015). Nat Mater.

[18] Stavrinidou E, Gabrielsson R, Gomez E, Crispin X, Nilsson O, Simon DT, Berggren M (2015). Sci Adv.

[19] Admassie S, Zhang F, Manoj AG, Svensson M, Andersson MR, Inganäs O (2006). Sol Energy Mater Sol Cells.

[20] Chen S, Lu BY, Xu JK, Qin LQ, Wang ZP, Duan XM (2013). J Appl Polym Sci.

[21] Stöcker T, Köhler A, Moos R (2012). J Polym Sci Part B Polym Phys.

[22] Zabrodskii AG, Zinov’eva KN (1984) Zh Eksp Teor Fiz 86:727

[23] Geretschläger M (1983). Nucl Instrum Methods Phys Res.

[24] Mayer M (1999). AIP Conf Proc.

