# Supplementary material for: Increase in electron scattering length in PEDOT:PSS by a triflic acid post-processing
Source: Monatsh Chem. 2017 Mar 31;148(5):871–7. doi: 10.1007/s00706-017-1973-1 (PMC5387017; doi:10.1007/s00706-017-1973-1)
Supplement: Supplementary file 1 — Supplementary material 1 (DOCX 796 kb) [file 706_2017_1973_MOESM1_ESM.docx]

# Supporting information

# Increase in electron scattering length in PEDOT:PSS by a triflic acid post-processing

D. Farka^1^, H. Coskun^1^, P.Bauer^2^, D. Roth^2^, B. Bruckner^2^, Petr Klapetek^3^ N. Serdar Sariciftci^1^ & P. Stadler^1^

1 Linz Institute of Organic Solarcells (LIOS), Institute of Physical Chemistry, Johannes Kepler University Linz, Altenberger Strasse 69, 4040 Linz, Austria.

2 Department of Physics, Atom and Surface Physics, Johannes Kepler University Linz, Altenbergerstr. 69, A-4040 Linz, Austria.

3 Department of Nanometrology, Czech Metrology Institute, Okružní 31, 63800 Brno, Czech Republic

Mgr. Dominik Farka, MSc

E-Mail: dominik.farka@jku.at


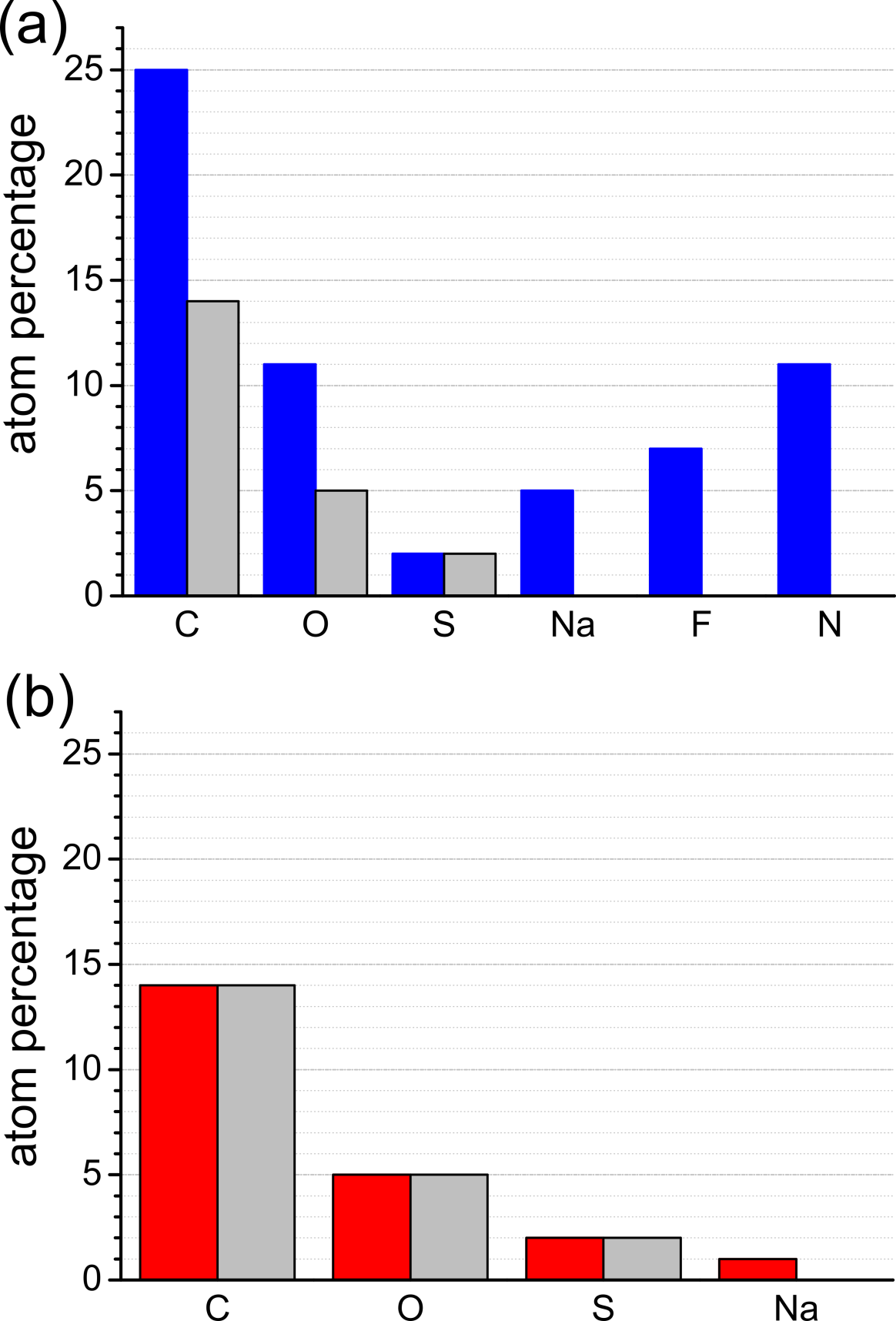


Figure 1: Results of elemental analysis by Rutherford Backscattering for PEDOT:PSS and PEDOT:PSS* (Density: 1.53 g/cm^3^) The grey area’s correspond to a stoichiometry of PEDOT:PPS = 1:1. Every deviation of the blue or red bar (respectively) from the amount shown in grey correspond to an impurity. Sodium, Fluorine, and Nitrogen correspond to impurities which come from the commercially obtained dispersions, just as are the excess amounts of carbon and oxygen. In PEDOT:PSS* thin-films a stoichiometric relationship was established and the amount of sodium was significantly reduced.


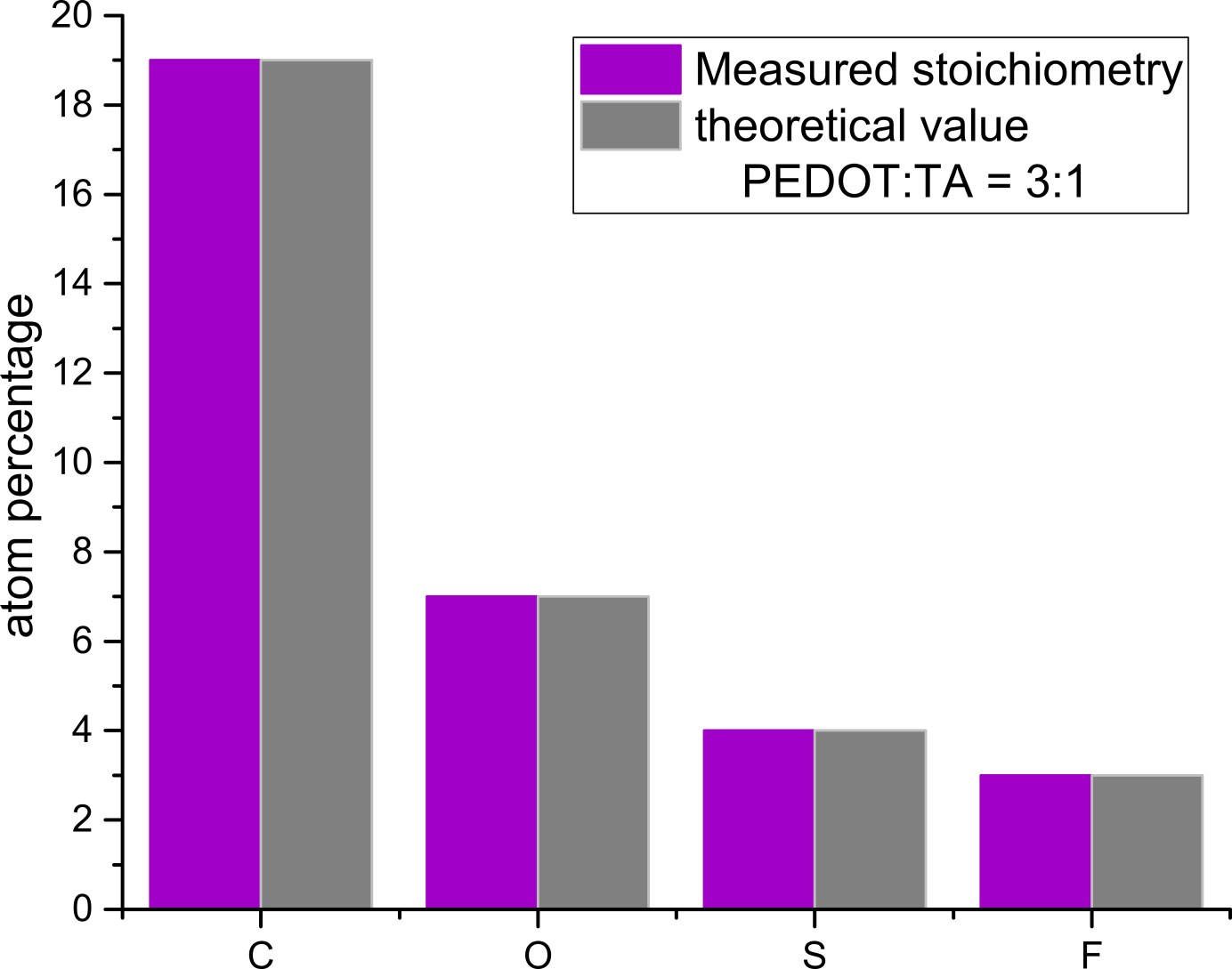


Figure 2: Results of elemental analysis by Rutherford Backscattering of PEDOT:TA. The measurement resulted in 3:1 ration of doping by triflic acid.

**Evaluation for the sample composition**

In RBS, individual elements *i* in a target material can be identified from the specific final energies of backscattered projectiles due to scattering kinematics, *E*_f,i_=*k*_i_*E*_0_. The kinematic factor *k* depends the scattering angle and the masses of projectile and target atoms, respectively [1]. Projectiles backscattered from heavier target atoms exhibit higher final energies compared to those backscattered from target atoms of lower atomic mass. Information on the composition, i.e., the areal densities in terms of atoms/cm^2^, is obtained from the backscattered yields due to from different elements. While the projectiles propagate in the sample, they are slowed down due to electronic stopping. Therefore, features in RBS spectra due to projectiles scattered at deeper layers, e.g., the substrate, are observed at energies lower than anticipated from scattering kinematics.

Evaluation of the compositions of the polymer samples was performed by comparing experimental spectra to corresponding simulations using the SIMNRA software package. SIMNRA permits to simulate RBS spectra for a target consisting of multiple individual layers of arbitrary composition within the single scattering approximation [2, 3]. In a first step, a sample composition according to theoretical stoichiometry was simulated. In the simulations, the areal densities were chosen such that the high energy edge due to scattering from the substrate was reproduced correctly. As an example, in Fig. 1 experimental and simulated spectra for 200 keV D^+^ scattered from PEDOT:PSS on Si are shown, for which a clear difference between simulation and experiment was observed. In particular, the experimental spectrum exhibits a higher yield between the signals of C and S, which may point towards the existence of additional elements with corresponding masses in the film. Note that H was considered in the simulation of the PEDOT:PSS film, due to scattering kinematics, however, backscattering from H is not possible in RBS. In a second step, the composition of the PEDOT:PSS film was fitted allowing for additional elements. For the PEDOT:PSS film best agreement between experiment and simulation was reached when in addition to H, C, O and S the elements N, F and Na were introduced, as depicted in Fig. 2. A similar procedure was applied for the PEDOT:PSS* and PEDOT:TA samples. The uncertainties of the final compositions range between ~ 10 % (PEDOT:PSS on Si) and ~ 20 % (PEDOT:TA on Al_2_O_3_), respectively. The larger uncertainties for the samples on Al_2_O_3_ are due to a non-vanishing signal due to scattering in the covering Au layer interfering with the S signal (roughness/multiple scattering effect due to acquisition at α=60°) and the small polymer signal superimposed on the substrate signal. Note that the lack of reliable data for electronic stopping in PEDOT samples also limits the accuracy of the evaluation.


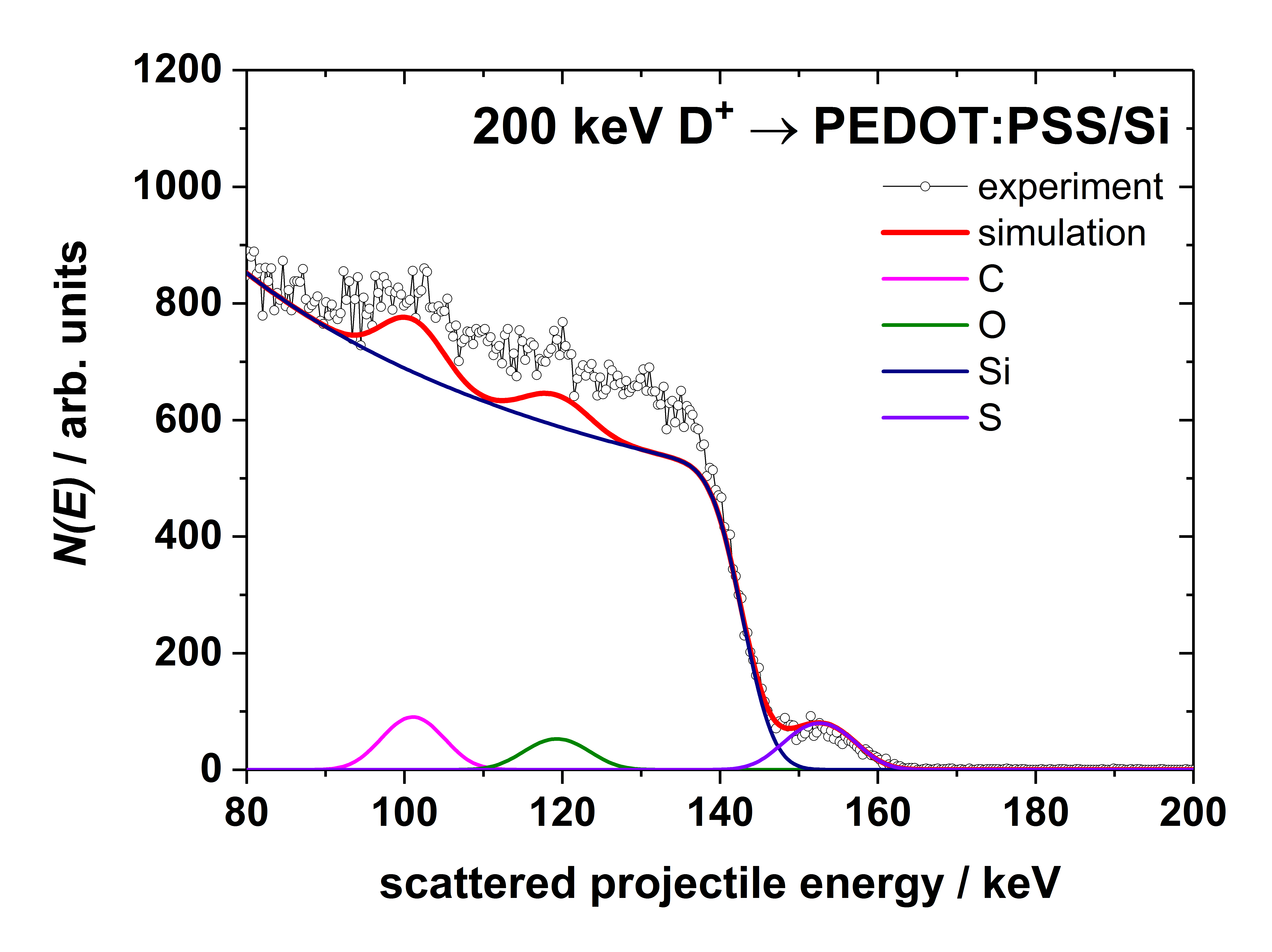


**Figure 3:** Experimental and simulated RBS energy spectra of 200 keV deuterons scattered from PEDOT:PSS on Si are shown. In the simulation a polymer composition according to the theoretical stoichiometry was chosen. Simulations of contributions due to scattering from different elements are also displayed.


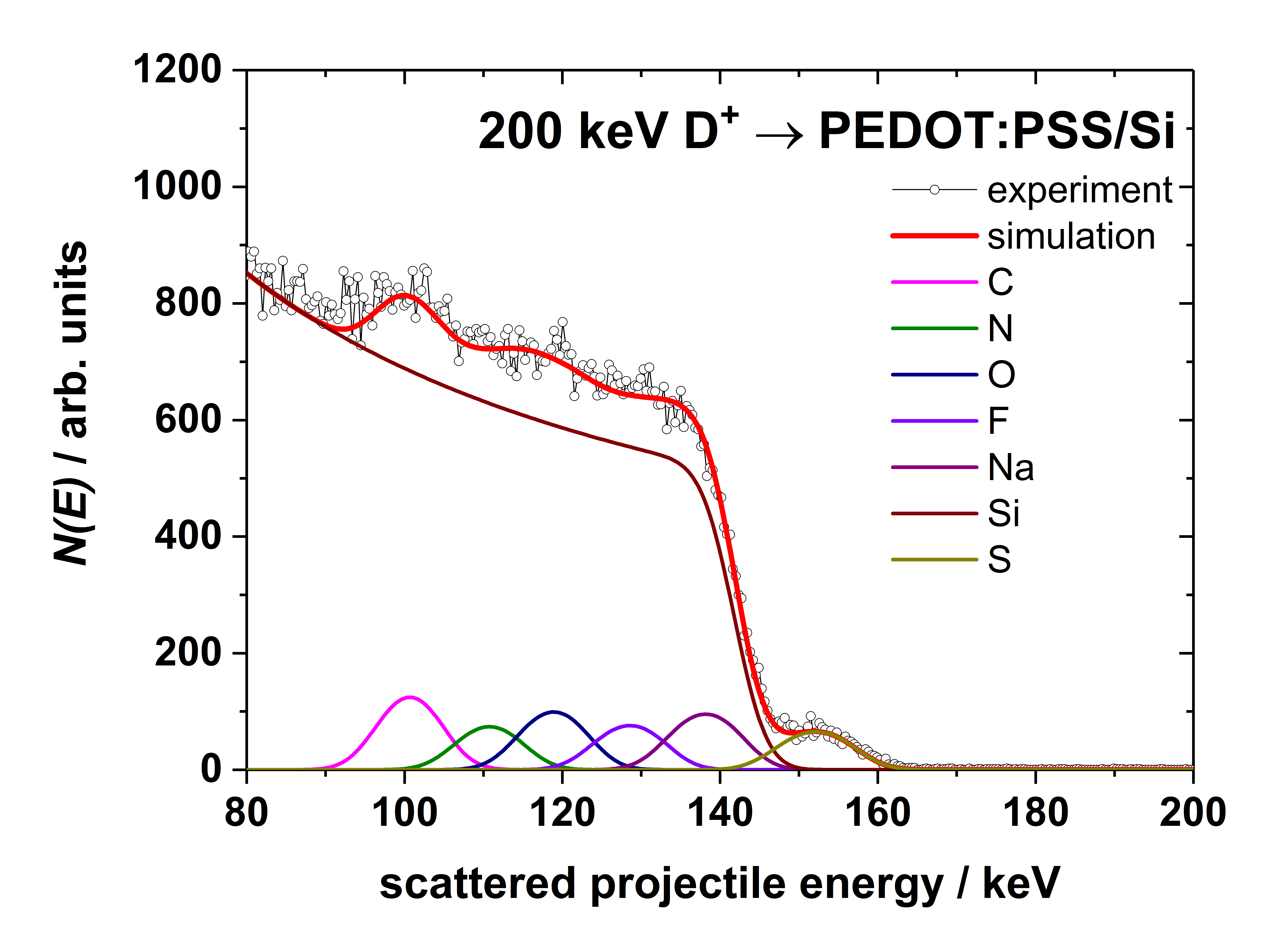


**Figure 4:** Experimental and simulated RBS energy spectra of 200 keV deuterons scattered from PEDOT:PSS on Si are shown. In the simulation the polymer composition was fitted by introducing new elements (N, F, Na). Simulations of contributions due to scattering from different elements are also displayed.

**References**

1. J.R. Tesmer MAN (1995) Handbook of Modern Ion Beam Materials Analysis. Materials Research Society

2. Mayer M (1999) SIMNRA, a simulation program for the analysis of NRA, RBS and ERDA. AIP Conf Proc 475:541–544. doi: 10.1063/1.59188

3. Geretschläger M (1983) A simple low-noise cryogenic preamplifier for silicon surface barrier detectors. Nucl Instruments Methods Phys Res 204:479–483. doi: 10.1016/0167-5087(83)90076-5
